# The Effect of Sodium Alginate-Coated Nano-Zinc Oxide on the Growth Performance, Serum Indexes and Fecal Microbial Structure of Weaned Piglets

**DOI:** 10.3390/ani14010146

**Published:** 2023-12-31

**Authors:** Xiao Xiao, Kai Guo, Jinsong Liu, Yulan Liu, Caimei Yang, Yinglei Xu, Bo Deng

**Affiliations:** 1 Key Laboratory of Applied Technology on Green-Eco-Healthy Animal Husbandry of Zhejiang Province, Zhejiang Provincial Engineering Laboratory for Animal Health and Internet Technology, College of Animal Science and Technology, College of Veterinary Medicine, Zhejiang Agricultural and Forestry University, Hangzhou 311300, China; 17367078635@163.com (X.X.); guokai980219@163.com (K.G.); xuyl@zafu.edu.cn (Y.X.); 2 Zhejiang Vegamax Biotechnology Co., Ltd., Anji, Huzhou 313300, China; vegamaxljs@163.com (J.L.); yulanflower@126.com (Y.L.); yangcaimei2012@163.com (C.Y.); 3 Institute of Animal Husbandry and Veterinary Science, Zhejiang Academy of Agricultural Sciences, Hangzhou 310021, China

**Keywords:** sodium alginate-coated nano zinc oxide, zinc oxide, piglets, growth performance, diarrhea, gut microbiota

## Abstract

**Simple Summary:**

The long-term overuse of zinc oxide by swine farms effectively alleviated the post-weaning diarrhea and improved the growth performance of piglets, whereas it also led to severe heavy metal residues and zinc emission pollution. Hence, finding traceable, safe, efficient and low-cost alternatives for zinc oxide is of great importance nowadays. In this study, sodium alginate-coated nano zinc oxide, a new type of zinc resource, was used and proved to be a potential alternative to traditional doses of zinc oxide, which showed beneficial effects on the growth performance, diarrhea rate, immune and antioxidant functions, zinc accumulation and excretion, short chain fatty acid concentration and the fecal microbial structure of weaned piglets. Our study laid a good foundation for the application of the novel coated nano zinc oxide in animals in the future.

**Abstract:**

High dose of zinc oxide (ZnO) could improve growth performance and alleviate disease status, whereas it caused serious environmental pollution and bacterial resistance. This study was to investigate whether low doses of sodium alginate-coated nano zinc oxide (saZnO), a new type of zinc resource, could serve as a potential alternative to pharmacological doses of traditional ZnO in weaned piglets. A total of 144 crossbred piglets were randomly allocated into three groups, including a basal diet without the addition of Zn (CON), a basal diet with 1600 mg Zn/kg from traditional ZnO (ZnO), and a basal diet with 500 mg Zn/kg from saZnO (saZnO). The experiment lasted for 28 days. The results showed that supplementing with ZnO and saZnO for 14 and 28 days significantly improved body weight (BW) and average daily gain (ADG) (*p* < 0.01) and markedly reduced the feed intake-to-gain ratio (F/G) (*p* < 0.05) and diarrhea rate. In addition, dietary ZnO and saZnO significantly increased the activities of the total antioxidant capacity (T-AOC) and alkaline phosphatase (ALP) (*p* < 0.01). Supplementing with saZnO also promoted the levels of superoxide dismutase (SOD), IgM and copper- and zinc-containing superoxide dismutase (Cu/Zn-SOD) in serum (*p* < 0.05), whereas a ZnO addition decreased the concentration of malondialdehyde (MDA) (*p* < 0.05), indicating the beneficial effect of Zn on antioxidant and immune functions. Piglets fed the ZnO diet showed higher serum Zn accumulations than those fed the CON and saZnO diets at d 28 (*p* < 0.01), and supplementing with ZnO and saZnO markedly contributed to Zn excretion in feces, especially in the ZnO diet (*p* < 0.01). Additionally, piglets fed the saZnO diet had greater valeric acid concentrations (*p* < 0.05) in their feces, while other short chain fatty acids (SCFAs) were not affected by different treatments (*p* > 0.05). Microbial alpha diversity was reduced in the saZnO group compared with the CON group (*p* < 0.05), while an obvious separation of microbial composition, the marker of beta diversity, was shown among the three groups (*p* < 0.05). At the genus level, six genera, including *Clostridium_sensu_stricto_1*, *Terrisporobacter*, f_Muribaculaceae, *Subdoligranulum* and *Intestinibacter*, were pronouncedly increased in the ZnO and saZnO groups (*p* < 0.05); another nine species were dramatically downregulated, such as f_Lachnospiraceae, f_Prevotellaceae, f_Butyricicoccaceae and f_Ruminococcaceae (*p* < 0.05). Finally, a functional analysis indicated that altered microbes significantly changed the “Metabolism” pathway (*p* < 0.05). These findings suggested that saZnO could act as a feasible substitute for ZnO to reduce Zn emission and enhance growth performance, antioxidant and immune functions, and to adjust the structure of gut microbiota in piglets.

## 1. Introduction

China is the largest pig-raising and pork-consuming country all over the world. With the rapid development of animal husbandry and the acceleration of intensive breeding processes, the early weaning of piglets has been an essential strategy for improving production efficiency for swine industries in recent years [1,2]. However, piglets, after weaning, are subjected to complex factors, including environment, pathogenic infection, shifts in nutrition and the immature development of digestive and immune systems, which frequently results in severe diarrhea and reduced feed intake, promoting morbidity and mortality, disturbed gut microbiota, and finally causing huge economic losses for the pig industry [3,4,5,6,7]. In the past few years, feeding pigs antibiotics was an effective mean to relieve post-weaning diarrhea and improving the growth performance of piglets [8,9]. However, a series of upcoming circumstances, such as bacterial resistance, antibiotic residues in animal products and environmental pollution, induced by the long-term excessive usage of antibiotics, has occurred [2,4,10]. Hence, finding safe and efficient antibiotic alternatives to promote growth and attenuate the diarrhea of weaned piglets is urgently required [11].

Over the past decades, zinc oxide (ZnO) has been globally used to relieve the post-weaning diarrhea and promote the growth performance of weaned piglets [12,13]. It was recognized as a potential substitute for antibiotics to relieve piglet diarrhea after weaning [11,14]. However, the long-term use of high-dose ZnO can also lead to a messy coat and pale skin in piglets, as well as animal toxicity [13]. More importantly, the majority of ZnO cannot be absorbed and is finally excreted through feces, causing Zn emission pollution, severe heavy metal residues and microbial resistance development [6,15]. In addition, the excessive emission of Zn from feces to the environment including to water and soil can ultimately endanger human health through the bio-concentration process [16]. In 2017, according to the announcement of the Ministry of Agriculture of China, the maximum amount of ZnO or alkaline zinc chloride (in terms of elemental Zn) in the feed of piglets is 1600 mg/kg [17]. Moreover, many other countries also limited the dosage of Zn due to severe environmental contamination, for example, in the EU, since 2017. Consequently, in the context of antibiotics being forbidden and Zn restrictions, developing traceable, safe, efficient and low-cost alternatives for ZnO is of great importance to pig farming nowadays.

Nano-ZnO, as an inorganic Zn, has a high bioavailability and is more effectively absorbed by animals than ZnO [11,13,18]. The literature has reported that dietary nano-ZnO and ZnO significantly improved average daily gain (ADG) and average daily feed intake (ADFI) and reduced fecal scores and the diarrhea incidence of piglets compared to the control group, while lower doses of nano-ZnO (500 mg/kg) showed the same growth as the pharmacological doses of ZnO (2500 mg/kg), suggesting that nano-ZnO could be a potential substitute for ZnO to improve growth performance and prevent diarrhea [18]. Nevertheless, nano-ZnO, similar to ZnO, was mostly digested into zinc iron in the acidic environment of the stomach and the minority of nano-ZnO finally took effect in the gut, thus reducing bioavailability and increasing emissions. The coating technology was widely used for drug delivery, which effectively improved the chemical stability and bioavailability, and allowed for the targeted release of substances. One study has inferred that under the protection of coating technology, ZnO can be safely retained to reach the intestine [19]. Previous studies have reported that coated-ZnO in low doses (500 mg/kg) could replace the traditional doses of ZnO (2000 mg/kg) to improve ADG and feed efficiency, alleviate diarrhea, enhance barrier function and maintain microbial homeostasis in weaned piglets [6]. Recently, coated nano-ZnO, which combined the advantages of nano-ZnO and coated-ZnO, was regarded as a potential suitable replacement for ZnO [18,19,20].

In our study, sodium alginate-coated nano-zinc oxide (saZnO) was synthesized and used. Accordingly, the objective of this experiment was to explore the effects of low doses of saZnO (500 mg/kg), as an alternative to traditional doses of ZnO (1600 mg/kg), on the growth performance, diarrhea rate, immune and antioxidant functions, Zn excretion and the fecal microbial structure of weaned piglets. 

## 2. Materials and Methods

All experimental protocols used in the animal experiment were approved by the Institutional Animal Care and Use committee of Zhejiang A & F University.

### 2.1. Experimental Products

The Zn purity of saZnO was 70%, which was synthesized and provided by Zhejiang Vegamax Biotechnology Company (Huzhou, China), and the supplementation of saZnO was of 500 mg Zn/kg in this study. ZnO was a common feed-graded source and was bought from Tysonzinc Company (Deyang, China), and the supplementation of ZnO was of 1600 mg Zn/kg, according to the guidelines of the Chinese Ministry of Agriculture, in this study.

### 2.2. Animals, Dietary Treatments and Experimental Design

A total of 144 crossbred weaned piglets (Duroc × Landrace × Yorkshire, 28 days old, from the same farm origin) with an initial body weight (IBW) of 7.95 ± 0.03 kg, were divided into three groups, six replicate pens per group and eight piglets per pen at random. The three treatment groups were as follows: a basal diet without any Zn source addition (CON, as the negative control), a basal diet + 1600 mg Zn/kg from ZnO (ZnO, as the positive control), and a basal diet + 500 mg Zn/kg from saZnO (saZnO). The whole experiment lasted for 28 days. The basal diet was formulated to meet the nutrient requirements according to the National Research Council (NRC 2012), as shown in Table 1. Piglets were obtained and reared at the experimental farm at Zhengxing Animal Husbandry CO., Ltd. (Hangzhou, China), and eight piglets per pen (4.8 m^2^ per pen and 0.6 m^2^ per piglet) were used for suitable pig density during the whole experiment. There was 1 automatic stainless steel nipple drinker and 1 cement feeder (6 slots) per pen. The study was conducted from May to June, 2023 in Linan, Hangzhou, Zhejiang Province. The conditions (temperature, humidity, et al.) of the farm was, in real-time, monitored and controlled, and all piglets were vaccinated following the farm’s routine vaccination program. All feed and water were available ad libitum.

### 2.3. Growth Performance and Diarrhea Rate

The weights of piglets and the feed consumption from each pen were recorded on d 1, 14, and 28 to evaluate the ADG, ADFI and feed intake-to gain-ratio (F/G), which are common indicators for growth performance. The diarrhea incidences of piglets for different phases were measured according to previous studies [21,22]. The diarrhea rate was calculated as the following: total number of pigs with diarrhea/(total number of pigs × number of experiment days) × 100.

### 2.4. Sample Collection

On d 28, one piglet from each pen (all 18 piglets) was selected to collect blood samples from the precaval vein, centrifuged at 3000× *g* for 15 min to obtain serum, and stored at −20 °C for further analysis. The same piglet from each pen was used to collect fresh fecal samples, which were immediately frozen in liquid nitrogen and stored at −80 °C for later analysis, including the contents of short chain fatty acids (SCFAs) and the structures of microbiota.

### 2.5. Serum Indexes

The levels of alkaline phosphatase (ALP) were measured by an automatic bio-chemical analyzer (Hitachi 7020, Hitachi High-Technologies, Tokyo, Japan). The concentrations of immunoglobulin M (IgM) and IgG in serum were determined by porcine-specific ELISA kits (Angle Gene Bioengineering Institute, Nanjing, China). The activities of antioxidant enzymes, including the total antioxidant capacity (T-AOC), glutathione peroxidase (GPx), superoxide dismutase (SOD), copper- and zinc-containing superoxide dismutase (Cu/Zn-SOD), catalase (CAT), and the content of malondialdehyde (MDA) in serum were detected by series kits according to the protocols (Angle Gene Bioengineering Institute, Nanjing, China).

### 2.6. Zn Concentrations in Serum and Fecal Samples

Frozen serum was thawed at room temperature and then vortexed until homogenized (~5 s). Then, 0.2 mL of serum samples was diluted 1:5 with deionized water, and wet-digested with 10 mL mixture of nitric acid and perchloric acid (9:1). Frozen feces were thawed at room temperature and an approximate 0.1 g of feces was diluted with deionized water, and wet-digested with 25 mL mixture of nitric acid and perchloric acid (9:1). Whole Zn concentration in serum and feces was measured by ICP-MS (Thermo Fisher Scientific, Cleveland, OH, USA).

### 2.7. SCFAs Concentrations in Feces

Fecal SCFAs were measured based on gas chromatography (GC, Agilent Technologies, Waldbronn, Germany) according to our previous study [22]. Briefly, 0.5 g of fecal samples were weighted and dissolved with 1.0 mL ultrapure water in a centrifuge tube (2 mL). The mixture was vortexed thoroughly for 30 s, kept in ice for 30 min, and subsequently centrifuged at 12,000 rpm for 10 min at 4 °C. A total of 1 mL of supernatant was collected and mixed with 200 μL of 25% phosphoric acid (*m/v*, 1:5) in a new centrifuge tube (2 mL). Then, the mixture was kept at 4 °C overnight. Subsequently, the samples were centrifugated at 4 °C, 12,000 rpm for 10 min, and the supernatant was collected and filtered using a 0.22 μm membrane and analyzed by GC.

### 2.8. Microbial Analysis Based on 16S rRNA Sequencing

Total microbial genomic DNA was extracted from the fecal samples using QIAamp DNA Stool Mini Kit (Qiagen, Hildern, Germany) according to the instructions. The V3–V4 region of the 16S rRNA gene was amplified using PCR. The primers used for amplification were 341 F: CCTAYGGGRBGCASCAG and 806R: GGACTACNNGGGTATCTAAT. The PCR products were purified and quantified. Subsequently, library quantification, normalization, and pooling were performed and we loaded the samples for MiSeq sequencing. Finally, the amplification primers were removed with cutadapt 2.4 and the trimmed reads were filtered for amplicon sequence variant (ASV) inference based on DADA2 1.12.1 algorithm. Sobs, chao1, Shannon, and Simpson’s indexes were calculated to reflect the bacterial diversity and richness based on the ASV table. Beta diversity was performed by principal coordinate analysis (PCoA) and visualized from complex data (a distinct clustering of microbiota composition) based on unweighted UniFrac metrics. Dissimilarity in community structure between samples was calculated by non-metric dimensional scaling (NMDS) based on Bray–Curtis metrics. Linear discriminant analysis with effect size (LEfSe) was used to identify microbes that were significantly changed. PICRUSt2 was applied to obtain functional predictions from the 16S rRNA data. Related analysis was performed using the free online platform of Majorbio Cloud Platform (www.majorbio.com, accessed on 26 August 2023).

### 2.9. Statistical Analysis

Statistical analysis was conducted using the SPSS 22 software (IBM company, New York, NY, USA) and GraphPad Prism 9.0 software (GraphPad software company, Boston, MA, USA). One-way ANOVA followed by Tukey’s multiple range tests were performed to access statistical significance. All the data were presented as mean ± SEM. *p* < 0.05 was regarded as statistically significant.

## 3. Results

### 3.1. Growth Performance and Diarrhea Rate

As shown in Table 2, the dietary supplementation of ZnO and saZnO for 14 and 28 days significantly increased the BW of piglets (*p* < 0.01), while no obvious difference in BW between the ZnO and saZnO groups was observed during the overall 28-day period (*p* > 0.05). The Dietary supplementation of ZnO and saZnO significantly increased the ADG during phase 1 (d 1 to 14) and throughout the trial (*p* < 0.01), whereas no difference in ADG was exhibited among the three groups during phase 2 (d 15 to 28) (*p* > 0.05). However, ADFI was not affected (*p* > 0.05) by dietary treatments during the whole period. F/G was markedly reduced in the ZnO group during phase 1 (d 1 to 14) and throughout the trial (*p* < 0.05) and in the saZnO group during phase 1 (d 1 to 14), phase 2 (d 15 to 28) and the overall experimental trial (*p* < 0.05). Importantly, piglets in the ZnO and saZnO groups exhibited reduced diarrhea rates (*p* < 0.05) compared to those in the CON diet during phase 1 (d 1 to 14) and phase 2 (d 15 to 28).

### 3.2. Biochemical Indices in Serum

As shown in Table 3, dietary ZnO and saZnO had no effect on the serum concentrations of GPx, CAT and IgG (*p* > 0.05). Compared to the CON group, ZnO supplementation significantly increased the activities of T-AOC and ALP (*p* < 0.01) and decreased the concentration of MDA (*p* < 0.05) in serum. Moreover, a saZnO addition markedly increased the concentrations of T-AOC, SOD, IgM, Cu/Zn-SOD and ALP (*p* < 0.05) in serum. Interestingly, a higher concentration of CAT and a lower concentration of Cu/Zn-SOD was observed in the ZnO group compared with the saZnO group (*p* < 0.05).

### 3.3. Zn Concentrations in Serum and Feces

As shown in Table 4, the ZnO group significantly increased serum Zn compared to CON and saZnO groups (*p* < 0.01), while no change in Zn concentration in serum was observed between the CON and saZnO groups (*p* > 0.05). In addition, the highest Zn excretion in feces was observed in the ZnO group, which differed markedly from those obtained in the CON and saZnO groups (*p* < 0.01). Furthermore, saZnO supplementation also significantly increased the level of Zn in feces compared to the CON group (*p* < 0.05), but dropped the level of Zn in feces comparing to the ZnO group (*p* < 0.05).

### 3.4. Fecal SCFAs Concentrations

As shown in Table 5, dietary ZnO and saZnO showed no influence on the concentrations of acetic acid, propionic acid, butyric acid, isobutyric acid, isovaleric acid and total SCFAs in the fecal samples (*p* > 0.05). However, saZnO supplementation significantly increased the level of valeric acid in the fecal samples compared with the CON group (*p* < 0.05), while ZnO addition showed an increased concentration of valeric acid (*p* > 0.05).

### 3.5. Microbiota Community

**Figure 1:**
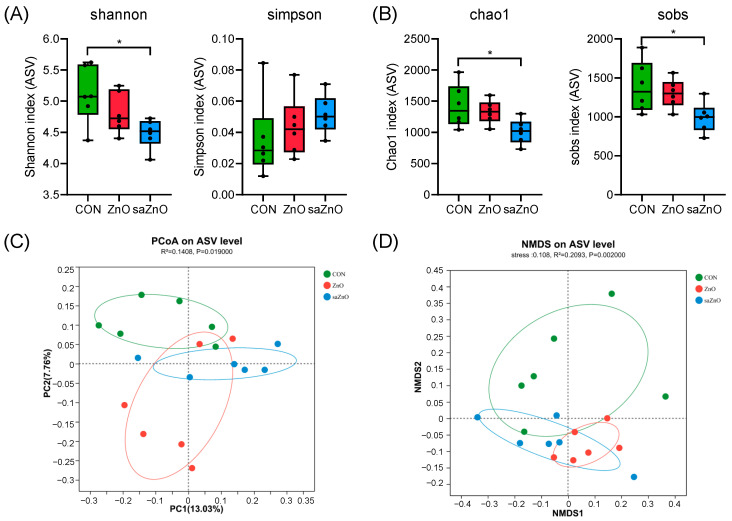
The alpha and beta diversities of microbiota in the fresh fecal samples of piglets. (**A**) Shannon and Simpson’s indexes. (**B**) Chao1 and Sobs indexes. (**C**) Principal coordinate analysis (PCoA) plot based on the unweighted UniFrac distances. (**D**) Non-metric dimensional scaling (NMDS) plot based on the Bray–Curtis metrics. CON, a basal diet without any Zn source addition; ZnO, a basal diet + 1600 mg Zn/kg from zinc oxide; saZnO, a basal diet + 500 mg Zn/kg from sodium alginate-coated nano-zinc oxide. * 0.01 < *p* < 0.05.

The microbial diversity of fecal samples was estimated and is shown in Figure 1. Shannon and Simpson’s indexes were used for microbial community diversity, while chao1 and Sobs were used for microbial community richness. The results in Figure 1A,B indicated that the saZnO group, showed significantly reduced Shannon, Chao1 and Sobs indexes compared to the CON group (*p* < 0.05), while no change in Simpson’s index was observed among the three groups (*p* > 0.05). Using the a above results, we inferred that dietary saZnO supplementation markedly reduced the alpha diversity of fecal microbiota. In addition, the PCoA plot based on the unweighted UniFrac distances showed a complete separation of microbial composition between the CON and saZnO groups (*p* < 0.05), while a partial overlap was exhibited between the ZnO group and the CON or saZnO group (Figure 1C). For further validation, the NMDS plot based on the Bray–Curtis metrics in Figure 1D also expressed totally different microbial compositions between the CON and saZnO groups (*p* < 0.05), which together confirmed that dietary saZnO supplementation changed the beta diversity of piglets.

**Figure 2:**
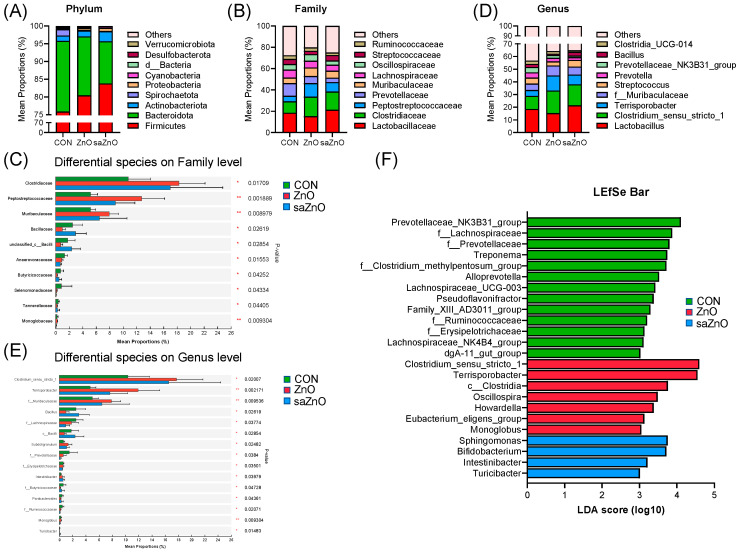
Relative abundances of fecal microbial communities at different taxonomic levels. (**A**) Top 10 species at the phylum level; (**B**) top 10 species at the family level; (**C**) differential species at the family level; (**D**) top 10 species at the genus level; (**E**) top 15 differential species at the genus level; (**F**) LEfSe linear discriminant analysis (LDA) score based on genus level. LDA score higher than three indicates a higher relative abundance. CON, a basal diet without any Zn source addition; ZnO, a basal diet + 1600 mg Zn/kg from zinc oxide; saZnO, a basal diet + 500 mg Zn/kg from sodium alginate-coated nano-zinc oxide. * 0.01 < *p* < 0.05 and ** *p* < 0.01.

For further analysis, the relative abundance of species at different taxonomic levels is shown in Figure 2. At the phylum level, Firmicutes and Bacteroidota were the major phyla among the three groups (Figure 2A). Firmicutes showed an upward trend and Bacteroidota showed a downward trend in the ZnO and saZnO groups compared with the CON group, whereas no obvious change in other species was observed in our study (Figure 2A). The relative abundance of the top 10 species at the family level is shown in Figure 2B. The dominant taxa among the three groups were Lactobacillaceae, Clostridiaceae, Peptostreptococcaceae, Prevotellaceae, Muribaculaceae and Lachnospiraceae, which accounted for approximate 60% of microbes. The analysis of differential species at the family level in Figure 2C further shows that the relative abundances of Clostridiaceae, Peptostreptococcaceae and Muribaculaceae were significantly enriched in the ZnO and saZnO groups compared to the CON group (*p* < 0.05), while the relative abundances of Butyricicoccaceae, Tannerellaceae and Monoglobaceae markedly dropped (*p* < 0.05). The top 10 species at the genus level indicated that *Lactobacillus*, *Clostridium_sensu_stricto_1*, *Terrisporobacter* and f_Muribaculaceae were the major taxa across all treatments (Figure 2D). Among the 15 differential genera, the mean proportions of 6 genera, including *Clostridium_sensu_stricto_1*, *Terrisporobacter*, f_Muribaculaceae, *Subdoligranulum* and *Intestinibacter*, were pronouncedly increased in the ZnO and saZnO groups compared with the CON group (*p* < 0.05) (Figure 2E). Another nine species were dramatically downregulated, such as f_Lachnospiraceae, f_Prevotellaceae, f_Butyricicoccaceae and f_Ruminococcaceae (*p* < 0.05) (Figure 2E). Furthermore, LEfSe plots based on the genus level showed that the relative abundances of *Prevotellaceae_NK3B31_group*, f_Lachnospiraceae, f_Prevotellaceae, *Treponema*, f_Clotridium_methylpentosum_group, *Alloprevotella* and *Lachnospiraceae_UCG-003* were predominant in the CON group; *Clostridium_sensu_stricto_1*, *Terrisporobacter*, *Oscillospira*, *Howardella*, *Eubacterium_eligens_group* and *Monoglobus* were the major species in the ZnO group, while species including *Sphingomonas*, *Bifidobacterium*, *Intestinibacter* and *Turicibacter* were markedly enriched in the saZnO group (Figure 2F).

**Figure 3:**
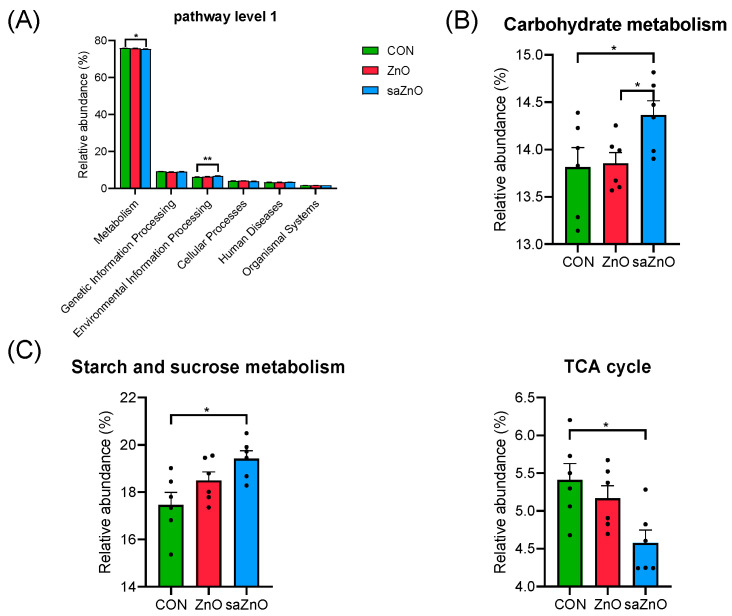
Functional analysis based on PICRUSt2. (**A**) Kyoto Encyclopedia of Genes and Genomes (KEGG) annotation of pathway level 1; (**B**) KEGG of differential pathways in level 2 of “Metabolism” in level 1; (**C**) KEGG of differential pathways in level 3 of “Carbohydrate metabolism” in level 2. CON, a basal diet without any Zn source addition; ZnO, a basal diet + 1600 mg Zn/kg from zinc oxide; saZnO, a basal diet + 500 mg Zn/kg from sodium alginate-coated nano-zinc oxide. * 0.01 < *p* < 0.05 and ** *p* < 0.01.

Previous studies have inferred that a change in microbial communities is accompanied by the functional transformation of flora [23]. Thus, Kyoto Encyclopedia of Genes and Genomes (KEGG) analysis was used to predict the altered pathway in our study. The results in Figure 3A showed that “Metabolism” was significantly reduced (*p* < 0.05), while “Environmental Information Processing” was pronouncedly increased (*p* < 0.01) in the saZnO group compared with that in the CON group in level 1. As “Metabolism” was the most abundant and differential pathway, we focused on it in pathway level 2. Only the relative abundance of “Carbohydrate metabolism”, belonging to “Metabolism” in level 1, was changed and enriched in the saZnO group compared to that in the CON group (*p* < 0.05) (Figure 3B). Thus, we further analyzed “Carbohydrate metabolism” in pathway level 3, among which “starch and sucrose metabolism” was increased and “TCA cycle” was decreased in the saZnO group compared to that in the CON group (*p* < 0.05) (Figure 3C).

## 4. Discussion

The long-term use of ZnO can effectively promote growth performance and attenuate diarrhea for post-weaning piglets [12,13]. However, excessive Zn accumulation leads to emission pollution, severe residues in meats and microbial resistance development [6,15]. Over the past few decades, numerous studies have reported that dietary ZnO and coated-ZnO supplementation improve growth performance and alleviate the post-weaning diarrhea of piglets [6,11]. In our study, the dietary supplementation of 1600 mg Zn/kg from ZnO and 500 mg Zn/kg from saZnO for 14 and 28 days both significantly increased the final BW and ADG and reduced F/G in weaned piglets, which was consistent with the literatures [11,18,24]. Contrary to the finding of Zhang [25], we did not find any impact of Zn addition on ADFI. What is more, the decreased diarrhea rate resulting from ZnO and saZnO supplementation was also in line with the notable effect of ZnO [13,14,20,26]. Previous studies have reported that coated-ZnO and nano-ZnO showed better effects on growth performance compared with ZnO supplementation in piglets [6,11]. In our study, saZnO at 500 mg Zn/kg exhibited a growth-promoting effect and reduced diarrhea rate comparable to that obtained by pharmacological doses of ZnO. Contrary to another finding [24], their results indicated that supplementation with 200 mg/kg of lipid-coated ZnO shows less ADG and gain: feed ratio than that of 2500 mg/kg of ZnO, which implies that coated-ZnO shows less beneficial effects than pharmacological ZnO. Thus, a proper concentration of Zn is essential for it to take effect. Collectively, our results demonstrated that saZnO shows greater promise of being an alternative to traditional ZnO. Nonetheless, the concrete mechanisms of saZnO supplementation on promoting growth performance and relieving diarrhea is still vague. 

In practice, post-weaning diarrhea, one of the most usual causes for poor growth and higher mortality, is usually accompanied by intestinal oxidative stress [2,12,27,28,29]. The complex system of antioxidant enzymes, including GPx, SOD and CAT et al., effectively protect the host against oxidative injury [28,30]. MDA, one of the final products of polyunsaturated fatty acid peroxidation, is the most commonly known product, whose levels can directly reflect the lipid oxidative injury and can be regarded as a reliable marker of antioxidant status [28,31]. Previous studies have reported that different Zn sources (different concentrations) increased the activities of SOD, GPx and T-AOC and reduced the level of MDA, and further influenced the antioxidant status of piglets [15,18,25,27,32]. In line with the previous literature, our results also showed that ZnO and saZnO supplementation significantly increased the activity of T-AOC. Moreover, saZnO supplementation markedly promoted the activity of SOD, while ZnO addition pronouncedly dropped the level of MDA in serum. No obvious change in GPx was observed among the three groups, and the activity of CAT showed an upward trend in the ZnO group and a downward trend in the saZnO group compared with the CON group. Immunoglobulins (Igs), including IgA, IgG, and IgM, are essential hallmarks of adaptive immunity [33]. Animal immune status is closely related to the levels of Igs in serum and tissues. Liu et al. [17] reported that hydrolysable tannins (HT) + ZnO supplementation significantly improve the levels of IgM and IgA in serum compared with the CON group. Peng et al. [34] and Zhang et al. [32] showed that porous ZnO and valine-chelated Zn (ZnVal) increased serum IgG. Another study also indicated that ZnO and nano-ZnO increased the serum concentration of IgA and decreased the concentration of IgM, but had no impact on IgG [35]. Similarly, our research indeed inferred that ZnO and saZnO showed no influence on serum IgG, whereas saZnO significantly improved the levels of IgM in serum. The above results demonstrated that saZnO showed a better promoting effect on antioxidant and immune functions than ZnO. 

The major Zn-containing enzymes, ALP and Cu/Zn-SOD, were regarded as biological indicators to evaluate the status of Zn in piglets [21,27,36]. Many studies have reported that different Zn resources pronouncedly increase the activities of ALP [14,21,34] and Cu/Zn-SOD [32]. In line with previous findings, saZnO and ZnO supplementation significantly increased the activity of serum ALP, while only saZnO markedly increased the level of Cu/Zn-SOD compared with the CON group, indicating a higher bioavailability of saZnO. As is known to all, the long-term overuse of Zn contributes to Zn emission and environmental pollution [6,15]. Thus, facilitating the absorption of Zn and reducing its excretion effectively is urgent. One study reported that diets supplemented with nano-ZnO and ZnO increased Zn retention in the serum, while nano-ZnO decreased the zinc excretion compared with conventional ZnO [35]. Compared with HZN (2250 mg Zn/kg from ZnO), CNZO (100 mg Zn/kg from coated-nano ZnO) decreased the levels of Zn in plasma and feces [20]. Not surprisingly, another study evaluated the effects of different forms of ZnO alternatives (Zn glycine chelate (ZnGly) and nano-ZnO) on Zn utilization in piglets; results showed that the blood concentration of Zn was significantly increased in the ZnO treatment group compared with that in the other treatment groups, and no change was observed between the CON and ZnO alternative (ZnGly/nano-ZnO) groups [26]. However, one previous study inferred that dietary ZnGly did not significantly influence the Zn distribution in the plasma [27]. Inconsistent with the above results, our findings show that ZnO supplementation significantly increased serum Zn compared to the CON group, while no obvious difference in Zn concentration in serum was expressed between the CON and saZnO groups. These findings were directly consistent with what has been found in a previous study [11]. In addition, our results indicated that ZnO markedly increased the Zn excretion in feces compared with the CON group. Although, saZnO supplementation reduced the Zn excretion in feces compared with the ZnO group, which was still higher than that in the CON group. A similar pattern of results was obtained in the literatures [11,21,26,35]. Thus, our results indicated that 500 mg/kg saZnO may improve Zn absorption and reduce fecal Zn excretion compared with pharmacological doses (1600 mg/kg) of conventional ZnO. Although the potential mechanism responsible for the effect of supplemental saZnO on Zn metabolism and utilization, other trace minerals’ excretion in weaned piglets remains to be further investigated.

The SCFAs, primarily derived from the microbial fermentation of dietary fiber through intestinal commensal bacteria, are beneficial for protecting gut epithelial integrity and barrier structure, regulating inflammation and mucosal immunity and maintaining intestinal homeostasis [37]. A previous study showed that Zn supplementation (ZnO and coated-ZnO) significantly increased the concentrations of acetic acid and propionic acid in the ileum and colon and dropped the concentration of butyric acid in the colon, but had no impact on butyric acid in the ileum [6], of which coated-ZnO showed a stronger effect than ZnO [6]. Another study indicated that dietary ZnO had higher concentrations of acetate and total SCFAs, while tetrabasic zinc chloride (TBZC) supplementation significantly increased the levels of acetate, propionate and total SCFAs in the fecal samples [21]. Inconsistent with the above results, our research demonstrated that dietary ZnO and saZnO showed no influence on the concentrations of acetic acid, propionic acid, butyric acid, isobutyric acid, isovaleric acid and total SCFAs in the fecal samples. In addition, no significant difference in SCFAs was observed between the ZnO and saZnO groups, which was in part similar to Zhang et al. [21]. Only saZnO supplementation significantly increased the level of valeric acid in the fecal samples compared with the CON group. Previous studies have inferred that valeric acid inhibits the release of inflammatory cytokines and the growth of pathogens, maintains barrier functions and microbial balance and further protects the host against injury [38,39,40,41]. Therefore, the increased valeric acid induced by saZnO supplementation was able to regulate intestinal health and facilitate growth performance.

Microbial composition and structure are essential for host homeostasis [6]. It is notable that microbial homeostasis is likely related to higher growth performance and less diarrhea [42]. Liu [19] and Wang [14] reported that no significant differences in alpha diversity, as indicated by Shannon, Simpson’s, ACE and Chao1 indexes, of the intestinal microbiota were found between treatment groups (ZnO vs. coated nano-ZnO; ZnO vs. SR-ZnO). Another study exhibited that, compared with the CON group, TBZC supplementation significantly increased the Ace and Chao indices of fecal microbes, whereas 1600 mg Zn/kg from ZnO supplementation was unable to promote microbial diversity [21]. Sun et al. [6] inferred that ZnO treatment reflected a higher Chao index to increase bacterial richness compared to the CON and coated ZnO groups. In our study, saZnO supplementation significantly reduced Shannon, Chao1 and Sobs indexes to reduce alpha diversity. No significant difference in alpha diversity was shown between the ZnO and saZnO group, which was similar than others [14,19]. Moreover, our results indicated that the microbial distribution distinctly separated among three groups, which was consistent with other reports [6,19,26]. We found that Firmicutes and Bacteroidota were the major phyla among the three groups, which was in line with previous findings [19,43,44]. In addition, increasing abundances of Clostridiaceae (*Clostridium_sensu_stricto_1*), f_Peptostreptococcaceae, f_Muribaculaceae, *Terrisporobacter*, *Subdoligranulum* and *Intestinibacter* were found in fecal samples, with the addition of different types and forms of Zn sources, which was found previously [44,45]. The potential pathogenic bacteria *Clostridium_sensu_stricto_1*, belonging to the Clostridiaceae family, was reported to produce SCFAs and promote intestinal mucus barrier adhesion to pathogens [46]. One study indicated that coated ZnO had a lower abundance of *Clostridium_sensu_stricto_1* than CON and ZnO groups [6], which was controversial with our study. Peptostreptococcaceae was reported to have a positive correlation with intestinal GPx activity and to further improve antioxidant capacity in weaned piglets [47]. Shang et al. [48] showed that probiotic Muribaculaceae presented a negative correlation with inflammation status in animals. Moreover, probiotic treatment significantly relieved diarrhea and increased the relative abundance of beneficial intestinal bacteria *Terrisporobacter* in mice [49]. Another study discovered that the abundance of *Subdoligranulum* showed a positive correlation with microbial richness, and a negative correlation with IL6 in humans [50], indicating a beneficial effect on the host. Partially, contrary to the above finding, we found ZnO supplementation to drop microbial alpha diversity and increase the abundance of *Subdoligranulum*. Liu et al. also reported that early supplementation with ZnO increased the relative abundances of *Subdoligranulum* compared with the CON group in pre-weaned dairy calves [51]. ZnO treatment raised the abundance of *Intestinibacter* [6], which was in line with our study. Another study demonstrated that two alternatives’ addition in dairy cows improved immune functions and the relative abundances of Lachnospiraceae, Monoglobaceae and Butyricicoccaceae in their feces [52], whereas our results showed that Zn supplementation enhanced the activities of antioxidant enzymes, but reduced the relative proportions of Butyricicoccaceae, Monoglobaceae and Lachnospiraceae. Butyricicoccaceae and Prevotellaceae, closely related to the levels of SCFAs [52], was inhibited by Zn addition in our work. Zinc sulfate supplementation significantly increased the abundance of Ruminococcaceae in dogs [53], which was inconsistent with our results. Pathogenic *Turicibacter* was reduced in a necrotic enteritis model and related to inflammation and immune cell balance [54]. *Turicibacter* was markedly enriched in the saZnO group, which was similar to previous studies [53]. *Bifidobacterium*, basically regarded as a probiotic, took effect in promoting metabolic health [55], which was enriched in the saZnO group. *Prevotellaceae_NK3B31_group*, members of the Prevotellaceae, could promote the production of SCFAs, thereby improving intestinal barrier integrity and functions in piglets [37]. However, Jiang et al. [56] demonstrated that the suckling piglets had higher lower abundances of the genus *Prevotellaceae-NK3B31_group* than that of the weaned piglets. A previous study showed that supplementation with different forms of Zn showed an increasing trend in the relative abundance of Prevotellaceae in the fecal samples of weaned piglets [21], while ZnO treatment raised the proportion of *Prevotellaceae_NK3B31_group* [6]. Controversially with previous data, *Prevotellaceae_NK3B31_group* was enriched in the CON group in our study, indicating a lower diarrhea rate of the ZnO and saZnO groups. Collectively, these were indicative for the promoting effect of Zn sources on intestinal health and host metabolism, especially the saZnO.

The robust change in microbial composition and structure is closely related to a functional transformation, and PICRUSt2 was used to predict the biological functions based on the KEGG database [23]. The results in our study showed that altered microbes were mainly involved in the “Metabolism” pathway. Microbiota, including *Clostridium_sensu_stricto_1,* Butyricicoccaceae, *Bifidobacterium*, *Prevotellaceae_NK3B31_group* and others participated in SCFAs’ production, nutrient absorption and intestinal functions [37,46,52,55], which were changed by different Zn supplementations in our study. 

Collectively, our study demonstrated that Zn supplementation significantly improved growth performance, as accessed by increased BW and ADG, as well as decreased F/G and diarrhea, increased antioxidant and immune functions, as indicated by higher concentrations of series enzymes and immunoglobulins and enhanced the activities of Zn-containing enzymes (ALP and Cu/Zn-SOD) and serum Zn accumulation and fecal Zn excretion. The results of the experiment provided clear support for the better effects of saZnO (low dose, 500 mg/kg) than ZnO (high dose, 1600 mg/kg). Furthermore, only the level of fecal valeric acid was pronouncedly increased by saZnO addition rather than ZnO, and the microbial structure, composition, and functions were markedly changed by Zn supplementation, especially saZnO. However, the major shortcoming of our study is that the pigs were not killed to collect intestinal contents and related tissues. Therefore, the effects of saZnO compared with ZnO on the nutrient digestibility, intestinal structure and integrity, Zn metabolism, barrier and immunological functions, intestinal microbial communities, and microbial-derived metabolites of weaned piglets need further investigation. In the future, more attention should be paid to investigate the concrete mechanism of saZnO in adjusting the intestinal health of weaned piglets.

## 5. Conclusions

In conclusion, the present research indicated that diets supplemented with 500 mg Zn/kg from saZnO showed more significant effects on growth performance, as indicated by increased BW and ADG, as well as decreased F/G and diarrhea compared with 1600 mg Zn/kg from traditional ZnO supplied for weaned piglets. Moreover, supplementation with saZnO and ZnO significantly improved the antioxidant and immune functions, as accessed by series enzymes and immunoglobulins compared with the CON group. Low doses of saZnO markedly reduced the Zn level in serum and Zn emission in feces compared with high doses of ZnO. Furthermore, only the level of valeric acid in the feces was increased in the saZnO group compared with the CON group. Significantly, saZnO mainly adjusted the diversity and structure of fecal microbiota, accompanied by increased probiotic bacteria and decreased pathogens. Altogether, saZnO showed better beneficial effects than ZnO in this study. Our research demonstrated that saZnO could act as a suitable alternative to traditional ZnO, which further provides a theoretical basis for the application of the novel coated nano-ZnO in animals.

## Figures and Tables

**Table 1:** Composition and nutrient levels of basal diets (dry matter basis, %).

Ingredients	Content, %	Nutrient Level	Content
Corn	55.00	DE, MJ/Kg	14.17
Wheat midding	3.50	CP, %	20.35
Phospholipid	2.00	Lys, %	1.34
Whey powder	5.00	Met + Cys, %	0.77
Extruded soybean	7.30	Thr, %	0.80
Soybean meal	18.50	Ca, %	0.95
Fish meal	5.00	Total P, %	0.65
Dicalcium phosphate	1.00	Available P, %	0.48
Limestone	1.10		
NaCl	0.10		
L-Lysine HCl	0.35		
DL-methionine	0.15		
Vitamin-mineral premix ^1^	1.00		
Total	100		

^1^ Supplied the following per kg of diet: vitamin A, 10,000 IU; vitamin D3, 400 IU; vitamin E, 10 mg; pantothenic acid, 15 mg; vitamin B6, 2 mg; biotin, 0.3 mg; folic acid, 3 mg; vitamin B12, 0.009 mg; ascorbic acid, 40 mg; Fe, 100 mg; Cu, 10 mg; Zn, 100 mg; Mn, 60 mg; I, 0.3 mg; and Se, 0.25 mg. Abbreviations: DE, digestible energy; CP, crude protein; Lys, lysine; Met, methionine; Thr, threonine; Ca, calcium; P, phosphorus.

**Table 2:** Growth performance and diarrhea rate.

Item ^1^	Treatments ^2^			*p* Value
	CON	ZnO	saZnO	
BW, kg				
d 1	7.96 ± 0.05	7.97 ± 0.06	7.91 ± 0.44	0.687
d 14	9.94 ± 0.87 ^b^	10.83 ± 0.11 ^a^	10.75 ± 0.20 ^a^	0.001
d 28	14.08 ± 0.20 ^b^	15.28 ± 0.24 ^a^	15.34 ± 0.33 ^a^	0.006
ADG, g				
d 1 to 14	141.43 ± 3.67 ^b^	204.29 ± 4.40 ^a^	202.86 ± 11.40 ^a^	0.000
d 15 to 28	295.71 ± 8.25	317.86 ± 10.03	327.86 ± 11.15	0.095
d 1 to 28	218.57 ± 5.85 ^b^	261.07 ± 6.87 ^a^	265.35 ± 10.70 ^a^	0.002
ADFI, g				
d 1 to 14	329.65 ± 13.40	350.48 ± 10.16	355.77 ± 21.40	0.480
d 15 to 28	650.60 ± 11.77	682.89 ± 14.39	686.25 ± 24.60	0.322
d 1 to 28	490.13 ± 10.89	516.69 ± 10.81	521.01 ± 21.81	0.334
F/G				
d 1 to 14	2.33 ± 0.06 ^a^	1.71 ± 0.02 ^b^	1.75 ± 0.01 ^b^	0.000
d 15 to 28	2.21 ± 0.04 ^a^	2.15 ± 0.02 ^ab^	2.09 ± 0.02 ^b^	0.050
d 1 to 28	2.25 ± 0.03 ^a^	1.98 ± 0.02 ^b^	1.96 ± 0.01 ^b^	0.000
Diarrhea rate, %				
d 1 to 14	33.30	6.30	8.30	
d 15 to 28	27.10	4.20	4.20	

^a, b^ means significant differences within a row (*p* < 0.05, *n* = 6). ^1^ BW, body weight; ADG, average daily gain; ADFI, average daily feed intake; F/G, feed intake to gain ratio. ^2^ CON, a basal diet without any Zn source addition; ZnO, a basal diet + 1600 mg Zn/kg from zinc oxide; saZnO, a basal diet + 500 mg Zn/kg from sodium alginate-coated nano-zinc oxide.

**Table 3:** Antioxidant enzymes and immunoglobulins in blood serum.

Item	Treatments			*p* Value
	CON	ZnO	saZnO	
T-AOC (U/mL)	22.02 ± 1.24 ^b^	26.93 ± 0.34 ^a^	28.49 ± 0.97 ^a^	0.001
GPx (μmol/L)	204.22 ± 9.26	218.51 ± 23.72	255.14 ± 28.71	0.276
SOD (U/mL)	50.80 ± 1.31 ^b^	63.13 ± 3.29 ^ab^	66.63 ± 5.88 ^a^	0.031
CAT (mmol/L)	260.44 ± 32.71 ^ab^	337.03 ± 24.43 ^a^	221.41 ± 28.41 ^b^	0.036
MDA (μmol/L)	3.90 ± 0.05 ^a^	3.68 ± 0.06 ^b^	3.79 ± 0.06 ^ab^	0.046
IgM (g/L)	0.59 ± 0.04 ^b^	0.64 ± 0.02 ^ab^	0.83 ± 0.09 ^a^	0.025
IgG (g/L)	4.21 ± 0.26	4.39 ± 0.34	4.79 ± 0.25	0.376
Cu/Zn-SOD (μg/L)	2.40 ± 0.05 ^b^	2.49 ± 0.06 ^b^	2.87 ± 0.03 ^a^	0.000
ALP (U/L)	235.67 ± 16.36 ^b^	276.33 ± 16.18 ^a^	295.00 ± 27.27 ^a^	0.004

^a, b^ means significant differences within a row (*p* < 0.05, *n* = 6). Abbreviations: T-AOC, total antioxidant capacity; GPx, glutathione peroxidase; SOD, superoxide dismutase; CAT, catalase; MDA, malondialdehyde; IgM, immunoglobulin M; IgG, immunoglobulin G; Cu/Zn-SOD, copper- and zinc-containing superoxide dismutase; ALP, alkaline phosphatase.

**Table 4:** Zinc (Zn) concentrations in serum and feces.

Item	Treatments			*p* Value
	CON	ZnO	saZnO	
Serum Zn (mg/L)	1.48 ± 0.23 ^b^	3.04 ± 0.43 ^a^	1.66 ± 0.19 ^b^	0.004
Fecal Zn (mg/kg)	235.91 ± 23.15 ^c^	2396.09 ± 113.43 ^a^	755.05 ± 58.90 ^b^	0.000

^a–c^ means significant differences within a row (*p* < 0.05, *n* = 6).

**Table 5:** Fecal short chain fatty acids (SCFAs) concentrations (ng/mg).

Item	Treatments			*p* Value
	CON	ZnO	saZnO	
Acetic acid	6359.47 ± 546.15	5570.28 ± 239.69	6680.13 ± 456.41	0.210
Propionic acid	3687.62 ± 319.12	3231.75 ± 216.74	3639.36 ± 171.85	0.373
Butyric acid	4388.08 ± 577.29	3716.04 ± 153.13	6417.06 ± 1345.34	0.097
Isobutyric acid	361.27 ± 59.04	355.99 ± 29.10	348.65 ± 34.90	0.979
Valeric acid	632.65 ± 76.63 ^b^	790.34 ± 65.17 ^ab^	942.53 ± 85.88 ^a^	0.038
Isovaleric acid	727.16 ± 117.14	689.97 ± 61.14	696.93 ± 65.27	0.948
Total SCFAs	16,156.24 ± 1332.94	14,354.38 ± 617.45	18,724.66 ± 1842.74	0.107

^a, b^ means significant differences within a row (*p* < 0.05, *n* = 6).

## Data Availability

The data in this article are available on request from the corresponding author.
